# CUMULATIVE ADVERSITY AND COGNITIVE FUNCTION AMONG OLDER ADULTS

**DOI:** 10.1093/geroni/igz038.3411

**Published:** 2019-11-08

**Authors:** Gabriella Dong, Mengting Li

**Affiliations:** Rutgers, The State University of New Jersey, New Brunswick, New Jersey, United States

## Abstract

The majority of studies on traumatic life events focus on posttraumatic stress disorder and depression, while less is known whether the cumulative exposure to traumatic events over the life course will deteriorate cognitive function. This study aims to investigate the association between lifetime traumatic events and cognitive function in an immigrant population. The data were drawn from the Population Study of Chinese Elderly in Chicago (PINE). Face-to-face interviews were conducted with a sample of 3,126 U.S. Chinese older adults in 2017-2019. Twelve types of traumatic events were assessed: physical assault, residential fires, sexual assault, miscarriage, abortion, imprisonment, being falsely accused, divorce, death of a loved one, being robbed, experiencing cancer, and being homeless. Cognitive function was measured through global cognition, episodic memory, working memory, processing speed, and Mini-Mental State Examination (MMSE). Linear regression was performed. In our sample, the maximum traumatic events experienced by one participant are eight types. Older adults who experienced one additional personal event were associated with higher global cognition (b=0.101, SE=0.012), episodic memory (b=0.130, SE=0.016), working memory (b=0.151, SE=0.034), processing speed (b=1.709, SE=0.178), and MMSE (b=0.124, SE=0.057), while controlling for age, gender, income, and education. In contrast with earlier studies, we identified the positive relationships between traumatic events and cognition. Older adults who had prior experience with stressful life events could demonstrate an advantage over those without such an experience. Further studies could investigate how individuals would respond to stressful life events, and how their resilience mechanism would promote cognitive function.

